# Development of an Intelligent Imaging System for Ripeness Determination of Wild Pistachios

**DOI:** 10.3390/s22197134

**Published:** 2022-09-21

**Authors:** Kamran Kheiralipour, Mohammad Nadimi, Jitendra Paliwal

**Affiliations:** 1 Mechanical Engineering of Biosystems Department, Ilam University, Ilam 69315-516, Iran; 2 Department of Biosystems Engineering, University of Manitoba, Winnipeg, MB R3T 5V6, Canada

**Keywords:** wild pistachio, ripeness, classification, machine vision, imaging processing

## Abstract

Rapid, non-destructive, and smart assessment of the maturity levels of fruit facilitates their harvesting and handling operations throughout the supply chain. Recent studies have introduced machine vision systems as a promising candidate for non-destructive evaluations of the ripeness levels of various agricultural and forest products. However, the reported models have been fruit-specific and cannot be applied to other fruit. In this regard, the current study aims to evaluate the feasibility of estimating the ripeness levels of wild pistachio fruit using image processing and artificial intelligence techniques. Images of wild pistachios at four ripeness levels were recorded using a digital camera, and 285 color and texture features were extracted from 160 samples. Using the quadratic sequential feature selection method, 16 efficient features were identified and used to estimate the maturity levels of samples. Linear discriminant analysis (LDA), quadratic discriminant analysis (QDA), and an artificial neural network (ANN) were employed to classify samples into four ripeness levels, including initial unripe, secondary unripe, ripe, and overripe. The developed machine vision system achieved a correct classification rate (CCR) of 93.75, 97.5, and 100%, respectively. The high accuracy of the developed models confirms the capability of the low-cost visible imaging system in assessing the ripeness of wild pistachios in a non-destructive, automated, and rapid manner.

## 1. Introduction

Wild pistachio is one of the most valuable forest trees grown in arid and semiarid mountains and altitudes [1]. The tree is a part of the Anacardiaceae family with various species, such as *Pistacia atlantica* and *Pistacia khinjuk* [2]. The economic trade value of pistachios worldwide was USD 3.85 billion in 2010–2011, which has increased to USD 9.57 billion in 2020–2021 [3], indicating the superior growth in the market demand for these tasty nuts. One of the pistachio varieties grown in the middle east is wild pistachio. 

Pistachios possess a unique flavor and color appropriate for use in cakes, ice cream, cookies, and several other food products [4]. Considering that around 30% of wild pistachio fruit is oil [5], it also has some industrial applications. The oil extracted from wild pistachio is highly stable [6] and can be used in colors, pesticides, glues, essences, papers, mineral oils, and other industrial applications [7,8]. Moreover, the wild pistachio resin is a traditional source of medicine for relieving abdominal pain, stomach pain, indigestion and stomach ulcers, asthma, eczema, sore throat, kidney stones, anti-diarrhea and astringent, anti-fever, antibacterial, and antiviral [9,10,11,12,13,14,15]. 

While wild pistachio has various applications, similar to other fruit, its composition changes during ripening, affecting its end-use and trade value. Therefore, it is critical to detect/harvest the fruit at different ripeness stages.

Over the past decade, and with the growing market demand for superior produce, the food industry has been actively looking for rapid, objective, non-destructive, and intelligent tools for the maturity detection of agricultural fruit and vegetables. In this regard, scholars have explored various tools such as near-infrared spectroscopy [16,17,18,19], or imaging techniques [20,21,22,23] to predict the ripeness levels of various agriproducts and/or to evaluate their quality parameters [24,25,26]. For example, the maturity of persimmon blueberry [27,28,29], tomato [30], apple [31,32], citrus [33], mulberry [34], and oil palm fruit [35] have been estimated using imaging and machine vision algorithms. Among the various spectral bands that can be explored in machine vision systems (such as visible, near-infrared, nuclear magnetic resonance, X-ray, and gamma-ray [36,37,38,39,40], the visible imaging range has been identified to be the most affordable. However, the prerequisite of implementing visible imaging systems for fruit ripeness estimation is the discriminability in the samples’ color characteristics at different ripeness stages. 

Despite several efforts on non-destructive fruit ripeness level estimations, our thorough literature review indicates that there has not been any previous effort on a smart assessment of the ripeness levels of wild pistachio. Considering that previously reported models on agri-products have been sample-specific, they could not be applied to other fruit, more specifically wild pistachio. Hence, to explore the feasibility of evaluating the maturity levels of wild pistachio, the present research aims to implement an intelligent computer vision approach to classify the wild pistachio fruit into four ripeness levels (i.e., initial unripe, secondary unripe, ripe, and overripe) using a low-cost visible imaging system. Such systems have already proven to revolutionize in-field and post-harvest quality management and preservation of cereal grains, legumes, oilseeds, and vegetables. 

## 2. Materials and Methods

**Figure 1:**
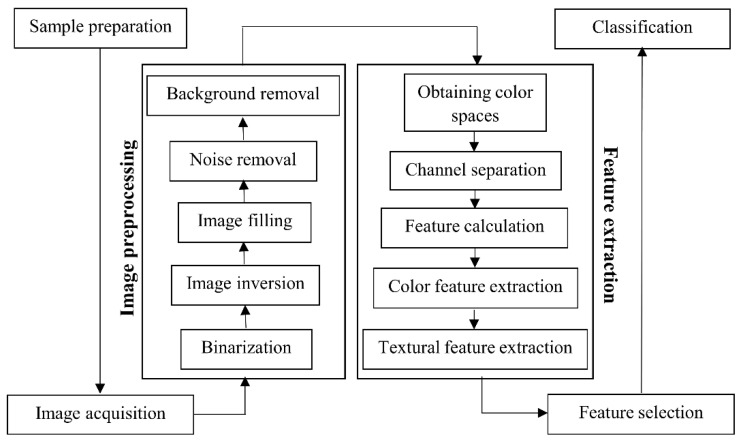
The operation steps in the ripeness classification of wild pistachio fruit.

Figure 1 shows the various steps of the proposed intelligent algorithm to identify the ripeness levels of wild pistachio fruit, which will be discussed in detail next.

### 2.1. Sample Preparation 

**Figure 2:**
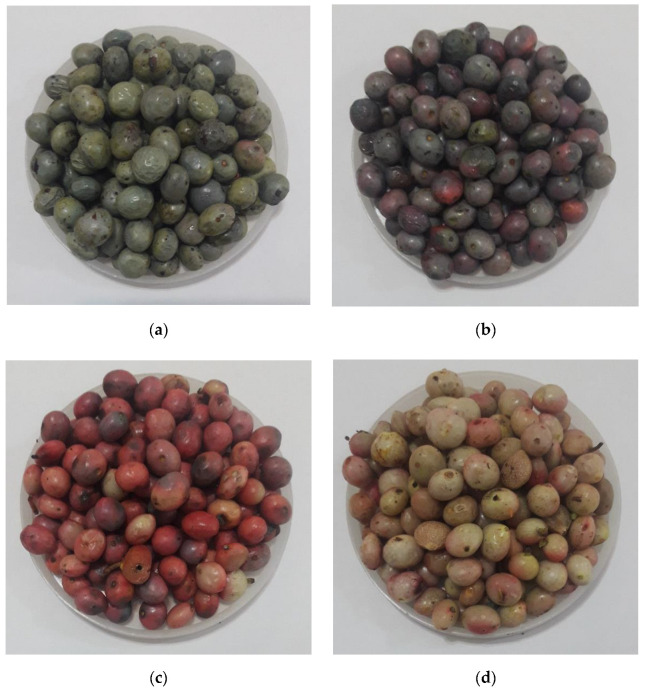
The separated wild pistachio samples, (**a**) overripe, (**b**) ripe, (**c**) secondary unripe, and (**d**) initial unripe.

The wild pistachio samples were acquired from the jungles of Ilam Province, Iran. Trained inspector panels established ground references for the four ripeness levels. It was found that the exterior colors of wild pistachio samples at initial unripe, secondary unripe, ripe, and overripe stages were dominantly close to white (white to cream and or pink), red (pink to red), blue, and green colors, respectively (see Figure 2). 

### 2.2. Image Acquisition

**Figure 3:**
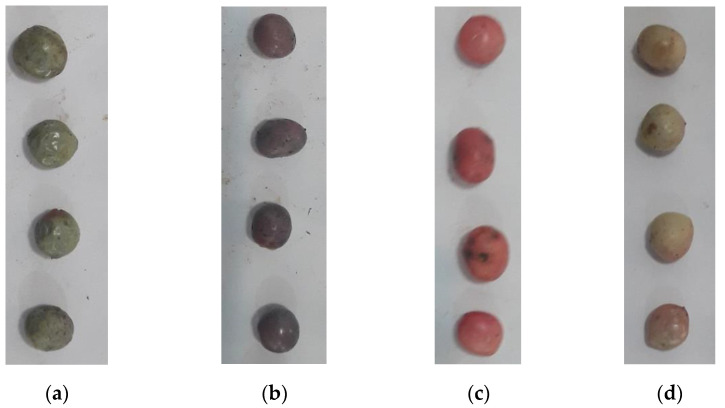
The image of wild pistachio samples, (**a**) overripe, (**b**) ripped, (**c**) secondary unripe, and (**d**) initial unripe.

To acquire images of individual samples, 160 wild pistachios (40 at each ripeness stage) were separately placed on an A4 white paper and imaged (Figure 3) using a Samsung Camera (resolutions: 13 MP, model J7, Samsung Corp., Seoul, Korea). The white background was chosen to simplify image segmentation (i.e., selecting a region of interest). The samples were imaged indoors in the lab under normal daylight.

### 2.3. Image Processing

As illustrated in Figure 1, the proposed image processing algorithm involved image pre-processing, feature extraction, and classification, which will be discussed next. Matlab Software (Version 2016a, Mathworks Inc., Waltham, MA, USA) was used for image analysis.

#### 2.3.1. Image Pre-Processing

Image pre-processing involved five main steps, including (1) conversion of an original image to a binary image, (2) image inversion, (3) applying erosion and dilation, (4) noise removal, and (5) background removal.

#### 2.3.2. Feature Extraction

Previous works have demonstrated the successful use of various color spaces and/or texture features in assessing the ripening levels of fruit [32,34,41]. In this work, we used a similar concept and extracted different color and texture features from each wild pistachio sample to identify their ripeness stage. The feature extraction included six different color spaces, viz. RGB, L* a* b*, I1I2I3, NRGB, CrCgCb, and HSV. The detailed definition of the color spaces mentioned above can be found elsewhere [42,43,44,45,46]. In addition, the gray level was obtained from RGB [47,48,49,50]. The data from 19 individual channels of the abovementioned color spaces were recorded, namely R, G, B, I1, I2, I3, L*, a*, b*, nr, ng, nb, cr, cg, cb, H, S, and V channels and gray levels. 

From the aforementioned image channels, 15 color and texture features were extracted, including minimum, mean, maximum, standard deviation, coefficient of variation, median, mode, skewness, kurtosis, homogeneity, covariance, contrast, correlation, entropy, and energy [47,51,52]. The detailed equations of the aforementioned features can be found elsewhere [42,45,46,47,51,52]. Overall, 15 × 19 = 285 features were extracted from each of the 160 samples.

#### 2.3.3. Feature Selection

The presence of redundant features could complicate the model development and data analysis. To this end, we used a quadratic sequential feature selection method (similar to [45,46,53] to identify and select optimum features for further analysis. The selected optimum features were used as inputs for the classification algorithms.

#### 2.3.4. Classification

Linear and quadratic discriminant analysis methods and artificial neural network [28,54,55,56,57] methods were employed to classify wild pistachios into four ripeness levels using the optimum features (see Section 2.3.3). Development of the classifier model was done in MATLAB Software. In the case of discriminant-based classifiers (i.e., LDA and QDA), the data were randomly divided into two sets, namely the training and test set, in a ratio of 80:20%. In the case of ANN-based classifiers, the data were randomly divided into three sets, namely the training, validation, and test set, in a ratio of 60:20:20%.

The ANN structure consisted of an input layer, a hidden layer and a target layer. The number of neurons in the input layer was equal to the number of the optimum features discussed above. The number of neurons in the target layer was set to the number of ripeness stages (=4). For the hidden layer, a varying number of neurons (between 2 and 20) were explored to identify the optimum structure. Similar to previous relevant studies [40,42,45], a tangent sigmoid activation function was used for the hidden layer, and a linear activation function was used for the target layer. The training of the ANN models (over epochs) was carefully monitored using the Matlab *plottrainstate* function, similar to [42]. The performance of ANN classifiers was compared based on the mean squared error (MSE) of validation set results, the correlation coefficient of the test data, and the correct classification rate (CCR) over the entire dataset [42,45].

The optimum ANN classifier was selected as a model with the highest CCR, the highest correlation coefficient, and the lowest mean squared error. The performances of discriminant-based classifiers (i.e., LDA and QDA) were examined using CCR and MSE measures. Ultimately, the CCR measure was utilized to compare the performance of LDA, QDA, and the optimum ANN model [42,45,46,53].

## 3. Results and Discussion

### 3.1. Image Pre-Processing

**Figure 4:**
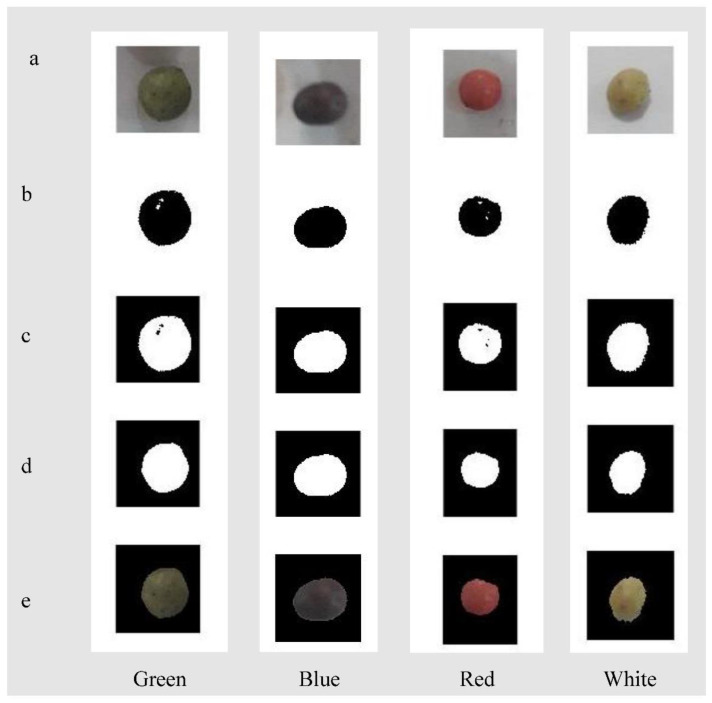
The result of different pre-processing steps, (**a**) original image, (**b**) black and white image, (**c**) reversing black and white image, (**d**) filling the image and removing the noises, and (**e**) removing the background from the image.

The result of image pre-processing is presented in Figure 4. In this step, the background and corresponding undesired shadows/components in each wild pistachio image were removed, and the final obtained image was used for the feature extraction step.

### 3.2. Feature Extraction

As mentioned in the material and methods section, 285 color and texture features were obtained from each wild pistachio image. Out of these, 16 features were identified as ‘optimum’ for classification, including the mean of B, skewness and kurtosis of L*, mean of b*, mean of Nr, mean and skewness of Ng, mean of Nb, mean of I2, mean and kurtosis of I3, mean of Cr, mean and skewness of Cb, mean of H, and mean of S channel.

Table 1 shows the average values of the aforementioned features for samples with different ripeness stages. The observed differences between the values within each row confirm the suitability of that feature for ripeness assessment. The provided features in Table 1 were used as the input of the classifier models. 

### 3.3. Discriminant Analysis Classifiers

Table 2 shows the confusion matrix of the LDA classifier model. The correct classification rate of the LDA classifier model was calculated to be 93.75%, with a mean squared error of 0.0625. It can be seen that only 1 out of 40 samples was misclassified as overripe, initial ripe, and secondary ripe stages. However, the ripe stage was misclassified in 7 out of 40 cases. 

The confusion matrix of the QDA classifier model is presented in Table 3. The correct classification rate of the QDA classifier was calculated to be 97.50%, with a mean squared error of 0.0250. It can be seen that the QDA outperformed LDA by yielding better accuracy and smaller error. In a set of 40 samples, the overripe, ripe, secondary ripe, and initial ripe stages were misclassified 2, 1, 1, and 0 times, respectively.

### 3.4. Artificial Neural Network Classifier

As indicated in Section 2.3.4, we also explored the suitability of various ANN structures to identify the ripeness stages of wild pistachios. Table 4 shows the performance of different ANN classifier structures.

**Figure 5:**
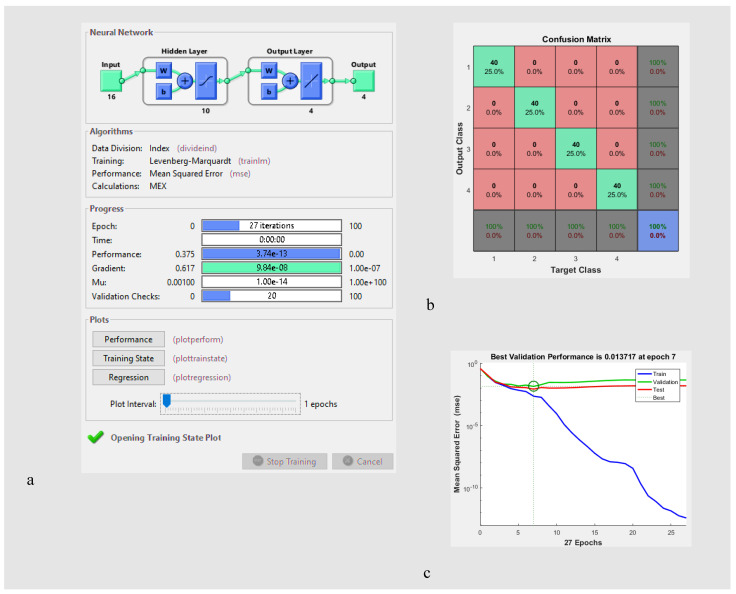
(**a**) The input, hidden, and output layers in the ANN classifier, (**b**) confusion matrix including correct classification rate of all data, and (**c**) mean squared error of validation data.

One can see that the best performance was achieved under a structure with 10 neurons (16-10-4), where the highest correct classification rate for the entire dataset was achieved (CCR = 100%) with the highest correlation coefficient of test data (r = 0.97779) and a relatively low mean squared error of validation data (MSE = 0.01372). Figure 5 shows the structure of the optimum ANN classifier, the corresponding confusion matrix (CCR = 100%), and the mean squared error curve (MSE = 0.01372). 

**Figure 6:**
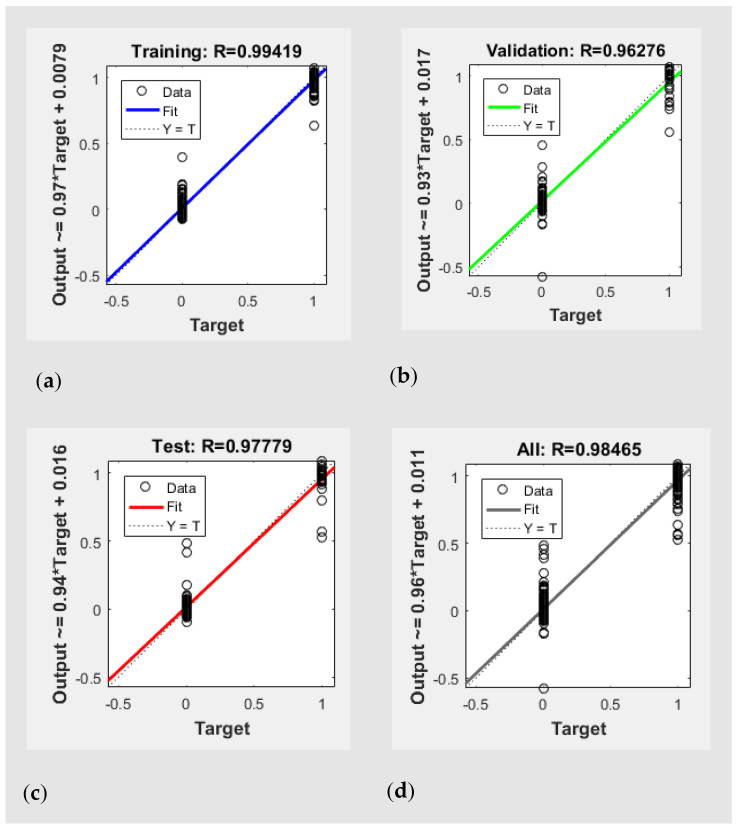
Correlation coefficient (R) of (**a**) training, (**b**) validation, (**c**) test, and (**d**) all data.

The regression lines and the correlation coefficients of the optimum ANN are shown in Figure 6. Herein, 0 and 1 represent the non-membership and membership of a sample for the desired class (see [42,45] for details). The correlation coefficients (R) of the optimum ANN model for training, validation, and test data sets were 0.99, 0.96, and 0.98, respectively. The correlation coefficient over the entire data was in excess of 0.98. One can see that rounding the predicted values to the closest binary values (0 or 1) can result in a perfect regression line (r = 1).

An alternative approach to using a shallow neural network to analyze our data could be state-of-the-art deep learning algorithms. However, the former approach was selected as it could offer reliable performance while needing lower computational time and power.

As mentioned in Section 1, to the best of our knowledge, there have not been any previous studies on smart assessment of the ripeness of pistachios. However, color imaging with LDA has been used by scholars to classify ripeness levels of banana (CCR = 98% using L* a* b* color space) [48], apricot (CCR = 90.4% using R, G, B channels, gray-scale, L*, a*, and b* color space) [58], and tomato (CCR = 81% using RGB color space) [59]. Similarly, QDA has been used by scholars to classify the ripeness levels of apricot (CCR = 92.3% using R, G, B channels, gray-scale, L*, a*, and b* color space) [58], and persimmon (CCR = 90.2% RGB + L* a* b* color space) [28]. ANN has also been implemented by scientists to classify the ripeness levels of mulberry (CCR = 96% using various color spaces [34]), banana (CCR = 96% using RGB color space) [60], tomato (CCR = 96% using L* a* b* color space) [30], and watermelon (CCR = 86.51% using YCbCr color space) [61]. Compared to the aforementioned works, our study confirms the reliability of visible imaging and image processing in identifying the ripeness stages of a new fruit (wild pistachio).

Future work in this area can involve examining the capability of the developed models on different pistachio cultivars and in orchard environments. Indeed, one should note that performing fruit segmentation in an orchard environment under variable light settings is a more challenging task (see [41]). Upon the development of appropriate segmentation algorithms, a modified model can be designed to be integrated into robots/drones to let stakeholders make efficient managemental decisions in the field. 

## 4. Conclusions

Wild pistachio is a fruit of high economic importance with various applications in the medicine and food industry. A non-destructive approach was developed to estimate the ripeness levels of wild pistachio using artificial intelligence and an image processing algorithm. Using linear discriminant analysis, quadratic discriminant analysis, and artificial neural network, classification accuracies of over 93% were obtained to classify wild pistachio images into four ripeness levels. The best performance was achieved using the artificial neural network, with an accuracy of 100%. The obtained results confirm the suitability of the proposed imaging algorithm combined with linear and non-linear classification techniques to characterize the ripeness levels of wild pistachios. However, further research is required to evaluate the capability of the developed model on various pistachio cultivars. Moreover, upon further research, the developed models can be integrated into harvesting robots to facilitate smart and efficient harvesting, grading, and handling of wild pistachios.

## Figures and Tables

**Table 1:** The mean value of the selected features of different groups of wild pistachios.

No.	Feature	Channel	Ripeness Stage.			
			Overripe	Ripe	Secondary Unripe	Initial Unripe
1	Mean	B	0.227351	0.246726	0.228051	0.318771
2	Skewness	L*	−0.24252	5.504441	23.80489	5.147078
3	Kurtosis	L*	1.076301	2.680921	4.210625	2.880944
4	Mean	b*	6.334246	2.581373	11.99416	9.648972
5	Mean	Nr	0.000231	0.000262	0.000459	0.000534
6	Mean	Ng	0.000225	0.000215	0.000232	0.000474
7	Skewness	Ng	21.3878	17.6356	5.723309	3.559942
8	Mean	Nb	0.000193	0.000213	0.000223	0.000421
9	Mean	I2	0.025847	0.023206	0.114415	0.063754
10	Mean	I3	0.008155	−0.01009	−0.05226	−0.00162
11	Kurtosis	I3	2.987824	2.948279	4.174406	3.320493
12	Mean	Cr	0.010891	0.030667	0.154239	0.052948
13	Mean	Cb	−0.0408	−0.01575	−0.07459	−0.07456
14	Skewness	Cb	−0.3614	−0.52836	0.269488	0.221479
15	Mean	H	0.143898	0.39319	0.247182	0.149145
16	Mean	S	0.183587	0.165916	0.500831	0.284045

**Table 2:** The confusion matrix of the LDA classifier model.

Predicted	Overripe	Ripe	Secondary Unripe	Initial Unripe
Actual				
Overripe	39	0	0	1
Ripe	7	33	0	0
Secondary unripe	0	0	39	1
Initial unripe	0	0	1	39

**Table 3:** The confusion matrix of the QDA classifier model.

Predicted	Overripe	Ripe	Secondary Unripe	Initial Unripe
Actual				
Overripe	38	2	0	0
Ripe	1	39	0	0
Secondary unripe	0	0	39	1
Initial unripe	0	0	0	40

**Table 4:** The performance of different ANN structures.

No.	Structure	Mean Squared Error of Validation	Correlation Coefficient Of Test Data	Correct Classification Rate of All Data
1	16-2-4	0.06441	0.80368	75.00
2	16-3-4	0.02591	0.93764	96.30
3	16-4-4	0.00020	0.88903	97.50
4	16-5-4	0.00861	0.93359	97.50
5	16-6-4	0.01584	0.91826	97.50
6	16-7-4	0.01014	0.85928	98.10
7	16-8-4	0.02546	0.92715	98.80
8	16-9-4	0.00204	0.92087	98.10
9	16-10-4	0.01372	0.97779	100.00
10	16-11-4	0.19684	0.94592	98.80
11	16-12-4	0.00748	0.95115	99.40
12	16-13-4	0.00391	0.89191	99.40
13	16-14-4	0.01795	0.90738	100.00
14	16-15-4	0.01710	0.95211	100.00
15	16-16-4	0.01770	0.94989	99.40
16	16-17-4	0.00897	0.94868	98.80
17	16-18-4	0.01328	0.94509	98.80
18	16-19-4	0.00779	0.96164	98.80
19	16-20-4	0.01447	0.92076	100.00

## Data Availability

The data that support the findings of this study are available from the corresponding authors, upon reasonable request.

## References

[1] Valipour P. Economical investigation of wild pistachio. The First National Seminar of Wild Pistachio (Green Pearl). Proceedings of the Natural Resources Research Center of Ilam Province.

[2] Zohary M. (1952). A monographical study of genus Pistacia. Palest. J. Bot..

[3] Statista (2022). Supply Value of Pistachios Worldwide from 2009/2010 to 2021/2022. https://www.statista.com/statistics/964028/production-value-of-pistachios-worldwide/.

[4] Statista (2022). Pistachio Market Worldwide and in the U.S.—Statistics & Facts. https://www.statista.com/topics/5158/pistachio-market/#topicHeader__wrapper/.

[5] Hosseinzadeh J., Tahmasebi M. Economic-Social Values of Wild Pistachio in Ilam Province. Proceedings of the Natural Resources Research Center of Ilam Province.

[6] Mousavian D.A., Niazmand R., Shariaee P. (2015). Investigation of the effect of coriander kernel oil, its non-detergent soaps in comparison with different coatings on the oil absorption of potato slices during the deep frying process. J. Innov. Food Sci. Technol..

[7] Fattahi M. Ecology of wild pistachio. The First National Seminar of Wild Pistachio (Green Pearl). Proceedings of the Natural Resources Research Center of Ilam Province.

[8] Heidarbeigi K., Ahmadi H., Kheiralipour K., Tabatabaeefar A. (2008). Some Physical and Mechanical Properties of Wild Pistachio (Pistachio Mutica L.). Am. Eurasian J. Agric. Environ. Sci..

[9] Tohidi M., Khayami M., Nejati V., Meftahizade H. (2011). Evaluation of antibacterial activity and wound healing of Pistacia atlantica and Pistacia khinjuk. J. Med. Plant Res..

[10] Panahi M., Barzegar H., Hojjati M. (2017). The effect of coriander gum essential oil on antimicrobial and antioxidant properties of starch edible film. Q. J. New Food Technol..

[11] Minaiyan M., Karimi F., Ghannadi A. (2015). Anti-inflammatory effect of Pistacia atlantica subsp. kurdica volatile oil and gum on acetic acid-induced acute colitis in rat. Res. J. Pharmacogn..

[12] Ghalem R.B., Mohamed B. (2010). Antimicrobial activity determination of the gum of Pistacia atlantica Desf. Afr. J. Microbiol. Res..

[13] Gourine N., Yousfi M., Bombarda I., Nadjemi B., Stocker P., Gaydon E.M. (2010). Antioxidant activities and chemical composition of essential oil of Pistacia atlantica from Algeria. Ind. Crops Prod..

[14] Hatamnia A.A., Abbaspour N., Darvishzadeh R. (2014). Antioxidant activity and phenolic profile of different parts of Bene (*Pistacia atlantica* subsp. *kurdica*) fruits. Food Chem..

[15] Hosseini F., Adlgostar A., Sharifnia F. (2013). Antibacterial activity of Pistacia extracts on Streptococcus mutans biofilm. Int. Res. J. Biol. Sci..

[16] Erkinbaev C., Nadimi M., Paliwal J. (2022). A unified heuristic approach to simultaneously detect fusarium and ergot damage in wheat. Meas. Food.

[17] Ali Shah S.S., Zeb A., Qureshi W.S., Arslan M., Ullah Malik A., Alasmary W., Alanazi E. (2020). Towards fruit maturity estimation using NIR spectroscopy. Infrared Phys. Technol..

[18] Nadimi M., Brown J.M., Morrison J., Paliwal J. (2021). Examination of wheat kernels for the presence of Fusarium damage and mycotoxins using near-infrared hyperspectral imaging. Meas. Food.

[19] Wang W., Paliwal J. (2007). Near-infrared spectroscopy and imaging in food quality and safety. Sens. Instrum. Food Qual. Saf..

[20] Cárdenas-Pérez S., Chanona-Pérez J., Méndez-Méndez J.V., Calderón-Domínguez G., López-Santiago R., Perea-Flores M.J., Arzate-Vázquez I. (2017). Evaluation of the ripening stages of apple (Golden Delicious) by means of computer vision system. Biosyst. Eng..

[21] Li X., Guillermic R.M., Nadimi M., Paliwal J., Koksel F. (2022). Physical and microstructural quality of extruded snacks made from blends of barley and green lentil flours. Cereal Chem..

[22] Nadimi M., Loewen G., Paliwal J. (2022). Assessment of mechanical damage to flaxseeds using radiographic imaging and tomography. Smart Agric. Technol..

[23] Sivakumar C., Chaudhry M.M.A., Nadimi M., Paliwal J., Courcelles J. (2022). Characterization of roller and Ferkar-milled pulse flours using laser diffraction and scanning electron microscopy. Powder Technol..

[24] Nadimi M., Sun D.W., Paliwal J. (2021). Recent applications of novel laser techniques for enhancing agricultural production. Laser Phys..

[25] Nadimi M., Sun D.W., Paliwal J. (2022). Effect of laser biostimulation on germination of wheat. Appl. Eng. Agric..

[26] Nadimi M., Loewen G., Bhowmik P., Paliwal J. (2022). Effect of laser biostimulation on germination of sub-optimally stored flaxseeds (*Linum usitatissimum*). MDPI Sustain..

[27] Tan K., Suk Lee W., Gan H., Wang S. (2018). Recognising blueberry fruit of different maturity using histogram oriented gradients and colour features in outdoor scenes. Biosyst. Eng..

[28] Mohammadi V., Kheiralipour K., Ghasemi Varnamkhasti M. (2015). Detecting maturity of persimmon fruit based on image processing technique. Sci. Hortic..

[29] Li H., Suk Lee W., Wang K. (2014). Identifying blueberry fruit of different growth stages using natural outdoor color images. Comput. Electron. Agric..

[30] Rafiq A., Makroo H.A., Hazarika M.K. (2016). Artificial Neural Network-Based Image Analysis for Evaluation of Quality Attributes of Agricultural Produce. J. Food Process. Preserv..

[31] Pourdarbani R., Sabzi S., Kalantari D., Karimzadeh R., Ilbeygi E., Arribas J.I. (2020). Automatic non-destructive video estimation of maturation levels in Fuji apple (*Malus Malus pumila*) fruit in orchard based on colour (Vis) and spectral (NIR) data. Biosyst. Eng..

[32] Sabzi S., Abbaspour-Gilandeh Y., García-Mateos G., Ruiz-Canales A., Molina-Martínez J., Arribas J. (2019). An Automatic Non-Destructive Method for the Classification of the Ripeness Stage of Red Delicious Apples in Orchards Using Aerial Video. Agronomy.

[33] Gan H., Lee W.S., Alchanatis V., Abd-Elrahman A. (2020). Active thermal imaging for immature citrus fruit detection. Biosyst. Eng..

[34] Azarmdel H., Jahanbakhshi A., Mohtasebi S.S., Rosado Muñoz A. (2020). Evaluation of image processing technique as an expert system in mulberry fruit grading based on ripeness level using artificial neural networks (ANNs) and support vector machine (SVM). Postharvest Biol. Technol..

[35] Septiarini A., Hamdani H., Rahmania Hatta H., Anwar K. (2020). Automatic image segmentation of oil palm fruits by applying the contour-based approach. Sci. Hortic..

[36] Vadivambal R., Jayas D.S. (2018). Bio-Imaging: Principles, Techniques, and Applications.

[37] Kheiralipour K., Ahmadi H., Rajabipour A., Rafiee S. (2018). Thermal Imaging, Principles, Methods and Applications.

[38] Jahanbakhshi A., Kheiralipour K. (2020). Evaluation of image processing technique and discriminant analysis methods in postharvest processing of carrot fruit. Food Sci. Nutr..

[39] Kheiralipour K., Ahmadi H., Rajabipour A., Rafiee S., Javan-Nikkhah M., Jayas D.S., Siliveu K., Malihipour A. (2021). Processing the hyperspectral images for detecting infection of pistachio kernel by R5 and KK11 isolates of *Aspergillus flavus* fungus. Iran. J. Biosyst. Eng..

[40] Kheiralipour K., Pormah A. (2017). Introducing new shape features for classification of cucumber fruit based on image processing technique and artificial neural networks. J. Food Process Eng..

[41] Sabzi S., Nadimi M., Abbaspour-Gilandeh Y., Paliwal J. (2022). Non-Destructive Estimation of Physicochemical Properties and Detection of Ripeness Level of Apples Using Machine Vision. Int. J. Fruit Sci..

[42] Azadnia R., Kheiralipour K. (2021). Recognition of leaves of different medicinal plant species using a robust image processing algorithm and artificial neural networks classifier. J. Appl. Res. Med. Aromat. Plants.

[43] Chaves-González J.M., Vega-Rodríguez M.A., Gómez-Pulido J.A., Sánchez-Pérez J.M. (2010). Detecting skin in face recognition systems: A colour spaces study. Digit. Signal Process..

[44] García-Mateos G., Hernández-Hernández J.L., Escarabajal-Henarejos D., Jaén-Terrones S., Molina-Martínez J.M. (2015). Study and comparison of color models for automatic image analysis in irrigation management applications. Agric. Water Manag..

[45] Khazaee Y., Kheiralipour K., Hosainpour A., Javadikia H., Paliwal J. (2022). Development of a novel image analysis and classification algorithms to separate tubers from clods and stones. Potato Res..

[46] Salam S., Kheiralipour K., Jian J. (2022). Detection of Unripe Kernels and Foreign Materials in Chickpea Mixtures Using Image Processing. Agriculture.

[47] Gonzalez R., Woods R. (2007). Digital Image Processing.

[48] Mendoza F., Aguilera J. Image Classification of bananas (Musa cavendish) during ripening based on appearance features. Proceedings of the Ninth International Congress on Engineering and Food.

[49] Lana M.M., Tijskens L.M.M., Van Kooten O. (2006). Effects of storage temperature and stage of ripening on RGB colour aspects of fresh-cut tomato pericarp using video image analysis. J. Food Eng..

[50] Kheiralipour K., Kazemi A. (2020). A new method to determine morphological properties of fruits and vegetables by image processing technique and nonlinear multivariate modeling. Int. J. Food Prop..

[51] Donis-González I.R., Guyer D.E., Leiva-Valenzuela G.A., Burns J. (2012). Assessment of chestnut (*Castanea* spp.) slice quality using color images. J. Food Eng..

[52] Kheiralipour K. (2012). Implementation and Construction of a System for Detecting Fungal Infection of Pistachio Kernel Based on Thermal Imaging (TI) and Image Processing Technology. Ph.D. Dissertation.

[53] Azadnia R., Kheiralipour K., Jafarian M. (2022). Evaluation of hawthorns maturity level by developing an automated machine learning-based algorithm. Ecol. Inform..

[54] Farokhzad S., Modaress Motlagh A., Ahmadi Moghadam P., Jalali Honarmand S., Kheiralipour K. (2019). Application of infrared thermal imaging technique and discriminant analysis methods for non-destructive identification of fungal infection of potato tubers. J. Food Meas. Charact..

[55] Kheiralipour K., Ahmadi H., Rajabipour A., Rafiee Javan-Nikkhah MJayas D.S., Siliveu K. (2015). Detection of fungal infection in pistachio kernel by long-wave near-infrared hyperspectral imaging technique. Qual. Assur. Saf. Crops Foods.

[56] Kheiralipour K., Ahmadi H., Rajabipour A., Rafiee S., Javan-Nikkhah M. (2015). Classifying healthy and fungal infected-pistachio kernel by thermal imaging technology. Int. J. Food Prop..

[57] Williams H.A.M., Jones M.H., Nejati M., Seabright MJBell J., Penhall N.D., Barnett J.J., Duke M.D., Scarfe A.J., Ahn H.S., Lim J.Y. (2019). Robotic kiwifruit harvesting using machine vision, convolutional neural networks, and robotic arms. Biosyst. Eng..

[58] Khojastehnazhand M., Mohammadi V., Minaei S. (2019). Maturity detection and volume estimation of apricot using image processing technique. Sci. Hortic..

[59] Polder G., Van Der Heijden GW A.M., Young I.T. (2002). Spectral image analysis for measuring ripeness of tomatoes. World Acad. Sci. Eng. Technol. Int. J. Comput. Inf. Eng..

[60] Krishnan R.P., Sofiah S., Radzi M. Color recognition algorithm using a neural network model in determining the ripeness of a banana. Proceedings of the International Conference on Man-Machine Systems (ICoMMS).

[61] Shah Rizam MS B., Farah Yasmin A.R., Ahmad Ihsan M.Y., Shazana K. (2009). Non-destructive watermelon ripeness determination using image processing and artificial neural network (ANN). World Acad. Sci. Eng. Technol. Int. J. Comput. Inf. Eng..

